# Clinically-Relevant Cutaneous Lesions by Nitrogen Mustard: Useful Biomarkers of Vesicants Skin Injury in SKH-1 Hairless and C57BL/6 Mice

**DOI:** 10.1371/journal.pone.0067557

**Published:** 2013-06-24

**Authors:** Neera Tewari-Singh, Anil K. Jain, Swetha Inturi, Carl W. White, Rajesh Agarwal

**Affiliations:** 1 Department of Pharmaceutical Sciences, Skaggs School of Pharmacy and Pharmaceutical Sciences, University of Colorado Denver, Aurora, Colorado, United States of America; 2 Department of Pediatrics, School of Medicine, University of Colorado Anschutz Medical Campus, Aurora, Colorado, United States of America; Roswell Park Cancer Institute, United States of America

## Abstract

A paucity of clinically applicable biomarkers to screen therapies in laboratory is a limitation in the development of countermeasures against cutaneous injuries by chemical weapon, sulfur mustard (SM), and its analog nitrogen mustard (NM). Consequently, we assessed NM-caused progression of clinical cutaneous lesions; notably, skin injury with NM is comparable to SM. Exposure of SKH-1 hairless and C57BL/6 (haired) mice to NM (3.2 mg) for 12–120 h caused clinical sequelae of toxicity, including microblister formation, edema, erythema, altered pigmentation, wounding, xerosis and scaly dry skin. These toxic effects of NM were similar in both mouse strains, except that wounding and altered pigmentation at 12–24 h and appearance of dry skin at 24 and 72 h post-NM exposure were more pronounced in C57BL/6 compared to SKH-1 mice. Conversely, edema, erythema and microblister formation were more prominent in SKH-1 than C57BL/6 mice at 24–72 h after NM exposure. In addition, 40–60% mortality was observed following 120 h of NM exposure in the both mouse strains. Overall, these toxic effects of NM are comparable to those reported in humans and other animal species with SM, and thus represent clinically-relevant cutaneous injury endpoints in screening and optimization of therapies for skin injuries by vesicating agents.

## Introduction

Among its multi-organ effects, one of the major concerns regarding exposure to chemical warfare agent, sulfur mustard (SM), is its toxic and inflammatory effects in skin, with delayed vesication in exposed regions [1]–[5]. SM has been used in numerous military conflicts since World War I [6]–[10], and SM effects on human skin are based mostly from Iranian soldiers and civilian population exposed to this agent during Iran-Iraq conflict [6]–[9]. Acute effects of SM on human skin occurring within 2–24 h include edema, erythema, blister, and bullae formation. The latter can burst and form a necrotic layer or eschar on the skin surface. The resulting ulcers or wounds are slow to heal and may lead to secondary infection [6], [11]–[13]. Blistering and necrosis of skin also could lead to long-term effects. Symptoms and signs may include dryness (xerosis), pruritis, desquamation, and hypo- and hyperpigmentation. Additional residual effects can include acne, cherry angiomas, scars, and chronic dermatitis [6], [11], [12], [14], [15]. These potentially debilitating effects can persist for many years.

The most valuable biomarkers of clinical relevance for assessing efficacy of treatments and interventions to mitigate skin injury due to SM are unknown. The clinical features of exposure to chemical warfare agents may be useful in diagnosis, treatment, and/or defining prognosis during the management of their exposure [16]. A number of animal species like guinea pigs, pigs and mice have been used to investigate mechanisms of SM-induced skin toxicity [17]–[21]. These models may parallel the clinical stages of SM exposure in humans. However, the observed vesication in these animal models is in the form of microblisters and gross vesication characteristic of SM exposure in humans is not evident [1], [22]. Besides SM, its analog, the primary vesicating agent nitrogen mustard (NM) that was stockpiled by several countries during World War II, also can pose a terrorist threat to civilians and warfare threat to military personnel [23], [24]. Due to limited access to the use of SM in laboratory settings, applicable quantitative clinical biomarkers have not been established. Therefore, we focused our studies on NM, a bi-functional alkylating agent like SM, whose effects are reported to be similar to SM [25], [26] and few studies are published with NM in human skin explants and mouse skin [25]–[30]. However, comprehensive studies identifying clinically-relevant cutaneous injury endpoints of NM-induced skin injury are not reported.

The present study reports the effects of NM exposure in both hairless SKH-1 and haired C57BL/6 mice, and identifies clinically relevant skin lesions, which were characterized during injury progression and quantitatively scored for the first time. Though hairless mice are a better study model for topical chemical exposures, there could be differences in uptake, absorption and skin injury responses to vesicating agents in hairless and haired mice. Furthermore, most genetic mice available for targeted mechanistic studies are on a C57BL/6 background. Therefore, both hairless and haired mice were used in this study. The clinical skin lesions observed with NM were comparable in both the strains, and these were similar to those reported in humans and other animal models with SM. Accordingly, the outcomes of present study provide valuable and relevant biomarkers for accelerated laboratory efficacy studies of therapies to treat both NM and SM, which pose warfare and terrorist threats to humans.

## Materials and Methods

### Ethics Statement

All animal work has been conducted according to relevant national and international guidelines, under an approved protocol by the Institutional Animal Care and Use Committee (IACUC) of the University of Colorado Denver.

### Animals and NM exposure

SKH-1 hairless and C57BL/6 mice (5–6 weeks old) were obtained from Charles River Labs (Wilmington, MA), housed under standard conditions, and acclimatized for one week before use in studies. All the NM exposures were done according to the prepared Standard Operating Procedure (SOP) by the IACUC of the University of Colorado Denver. Animals were monitored regularly for any abnormal changes in their health or behavior, and veterinarian was consulted when required. Three-days prior to NM exposure, C57BL/6 mice were shaved using clippers. Both SKH-1 hairless and shaved C57BL/6 mice (5 per group) were untreated, exposed topically to 200 µL acetone alone or 3.2 mg NM (Sigma-Aldrich Chemical Co., St. Louis MO)/mouse in 200 µL acetone (260 µg/cm^2^ on defined area of mouse dorsal skin) for 12 h, 24 h (1 day), 72 h (3 days) and 120 h (5 days). This dose selection was based on reports of human and animal exposure to higher dose of SM, as well as NM studies showing skin injury, vesication, and both acute and chronic effects of SM [4], [20], [31]. Acetone was used as a vehicle because it enhances skin permeability to these compounds [32], [33]. Using an electronic digital caliper (Marathon Inc. Belleville, ON, Canada), the dorsal skin bi-fold thickness was measured (mm) at all time-points following NM exposure. The NM-caused appearance of any changes or lesions and their progression (e.g. blistering, edema, erythema, pigmentation changes, xerosis, wounding, etc., which are mainly also related to SM skin exposure) on the exposed skin were pictured and evaluated in terms of scores corresponding to the injury or lesion observed as detailed in Tables 1, 2, and 3. The NM-induced injury lesions at all time-points were observed and scored by three individuals independently for each mouse (5 mice per group till 72 h in both control and NM-exposed groups, and 2-3 mice in NM-exposed group and 5 mice in the control group at 120 h following NM exposure), and an average was then calculated. Also, at study end points, either mortality in mice was noted or the live mice were euthanized and dorsal skin was collected for desired studies.

**Table 1:** Progression of NM-induced skin injury in terms of clinical lesion development in SKH-1 hairless and C57BL/6 mice.

Arbitrary score	NM-induced clinical lesion development
**1**	No significant difference from the normal skin
**2**	Moderate to severe edema and erythema
**3**	Moderate to severe edema and erythema, presence of some wounded slightly scratched areas on the skin
**4**	Moderate to severe edema and erythema in patches, and swollen skin with appearance of small and large microblisters
**5**	Moderate to severe edema and erythema, swollen skin with appearance of large and small microblisters, and some areas with pigmentation changes
**6**	Defined edema and moderate erythema, appearance of large microblisters, some areas with pigmentation changes, few wounds on skin with red broken skin
**7**	Defined edema and moderate erythema, appearance of large microblisters with lesser swollen skin, increase in pigmentation changes (moderate), and more wounded skin with larger and more cut skin areas with some dry patches
**8**	Moderate to slight edema and erythema but thick wrinkled skin, decrease in appearance of large microblisters, increased and more distinct pigmentation changes, increased wounding with cut and exposed skin areas, dry wrinkled skin (xerosis) and some small areas of peeling skin (desquamation)
**9**	Moderate to slight edema and erythema but thick leathery wrinkled skin, decrease in appearance of microblisters, increased and more distinct pigmentation changes, increased wounding incidence and area and some broken wet exposed skin, dry scaly peeling skin (xerosis and desquamation)

**Table 2:** NM-induced skin edema and erythema in SKH-1 hairless and C57BL/6 mice.

Arbitrary score	Skin edema and erythema	Appearance of microblisters
**1**	No significant edema or erythema as compared to normal control skin	No significant appearance of microblisters as compared to normal control skin
**2**	Moderate and well defined edema and erythema	Swollen skin with appearance of small and large microblisters
**3**	Moderate to severe edema and erythema	Fluffy skin with appearance of large microblisters
**2.5**	Severe edema and moderate erythema	Larger appearing microblisters but with lesser swollen skin (compressed)
**1.5**	Moderate to slight edema and erythema but thick leathery wrinkled skin	Decrease in appearance of large microblisters

**Table 3:** NM-induced skin wounding and pigmentation changes, and dry and scaly skin (xerosis) in SKH-1 hairless and C57BL/6 mice.

Arbitrary score	Skin wounding and pigmentation changes	Dry and scaly skin
**1**	No significant skin wounding or pigmentation changes as compared to normal control skin	No significant increase in dry peeling, scaly skin as compared to normal control skin
**2**	Presence of some wounded small scratches on the skin	Some dry patches on the skin
**3**	Few wounds on skin in patches with red broken skin and some areas with pigmentation changes	Large areas of dry wrinkled skin
**4**	More wounded skin with larger and more cut areas an, increase in pigmentation changes (moderate)	Leathery, wrinkled and dry scaly peeling skin with broken skin appearance
**5**	Wounding and more peeled skin areas with wet skin, and more distinct pigmentation changes	-

### Statistical analysis

Results were analyzed by one-way analysis of variance (one-way ANOVA) to define statistically significant differences between control and treated groups, followed by Tukey or Benferroni t-test for multiple comparisons (SigmaStat 2.03). Differences were considered significant if the *p* value was ≤ 0.05. Data are presented as mean ± standard error of mean (SEM).

## Results

### NM exposure induces skin toxicity causing clinical lesions and an increase in skin bi-fold thickness in SKH-1 and C57BL/6 mice

#### Skin injury progression and evaluation of clinical lesions

**Figure 1:**
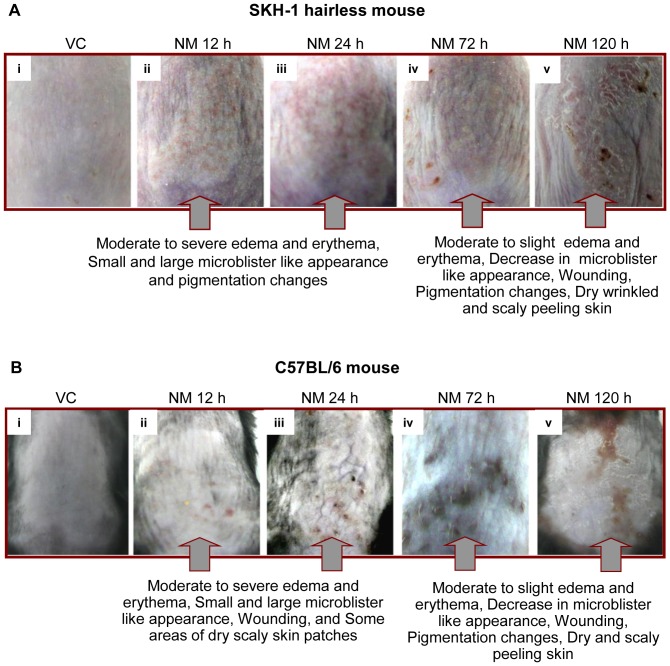
Effect of time-dependent NM exposure on the cutaneous toxicity and clinical sequelae of injury progression in SKH-1 hairless (A) and C57BL/6 (B) mice. Dorsal skin of mice was exposed topically to either 200 µL of acetone or NM (3.2 mg) in 200 µL acetone, and animals were observed for NM-induced clinical effects/lesions on exposed skin tissue. Pictures were taken and the visible injury lesions were compared between SKH-1 hairless (A) and C57BL/6 (B) mice. VC, vehicle control.

**Figure 2:**
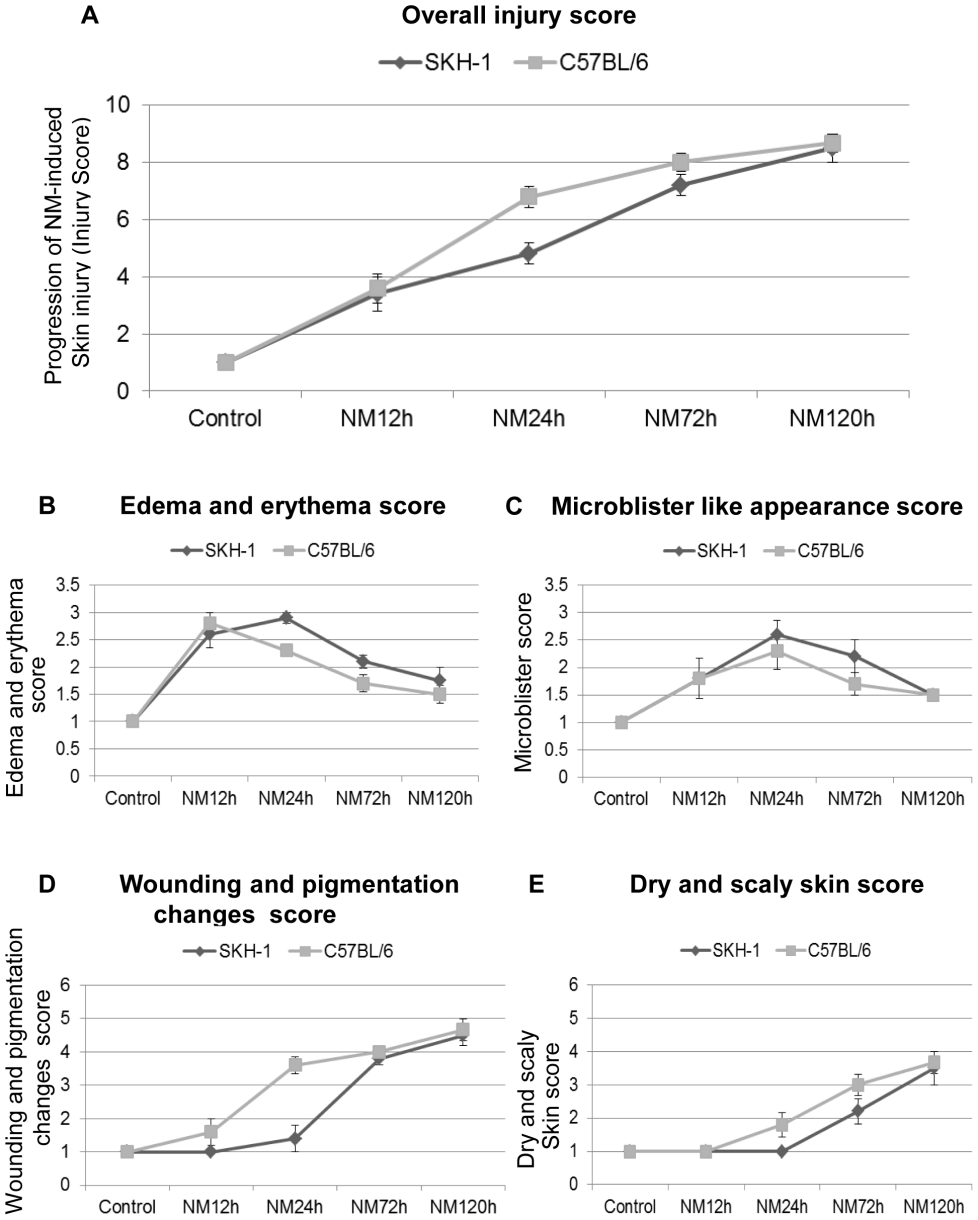
Quantitative evaluation of the cutaneous lesions and clinical sequelae of injury progression following time-dependent NM exposure in SKH-1 hairless and C57BL/6 mice. Dorsal skin of mice was exposed topically to either 200 µL of acetone or NM (3.2 mg) in 200 µL acetone. After NM exposure, as a function of time, an overall injury progression (A), clinical lesions namely edema and erythema (B), appearance of microblisters (C), wounding and pigmentation changes (D), and scaly and dry (xerosis) skin (E) were scored. These scores were compared in both SKH-1 hairless and C57BL/6 mice as detailed under Materials and Methods, and recorded in Tables 1, 2, and 3. Data presented are mean ± SEM of 5 animals in each group; VC, vehicle control.

Following NM exposure, comparable skin injury lesions were observed in both mouse strains; however, there were slight differences in their appearance and progression with time from 12–120 h post-NM exposure as shown in representative illustrations (Fig. 1, Table 1). These lesions were evaluated by scoring overall injury progression and specific clinical lesions in both the mouse strains (Fig 2, Tables 1, 2, and 3).

An inflammatory response to toxic exposure leads to reddening of skin due to an increase in the immune response cells (erythema) and accumulation of fluid (edema); these are mostly accompanied with cellular hyperplasia [34]. Within 12 h of NM exposure, in addition to moderate edema and erythema, small yellowish microblisters appeared with swollen appearance in SKH-1 hairless mice skin, which increased with time and were most evident at 24 h of exposure (Fig. 1A ii, Fig 2A–C, Tables 1 and 2). However, in C57BL/6 mice, after 24 h of NM exposure, only moderate and more clearly demarcated edema and erythema were noted, appearing less severe than that in SKH-1 hairless mice by this time (Fig. 1A and B ii and iii, Fig 2 A–C, Tables 1 and 2). Overall, NM-induced skin injury progression scores were: 3.4 ±0.60 and 3.6±0.51 at 12 h, and 4.8±0.37 and 6.8±0.37 at 24 h of NM exposure in SKH-1 hairless and C57BL/6 mice, respectively (Fig. 2A, Table 1). At 24 h of NM exposure, maximum edema and erythema scores were 2.9±0.10 and 2.4±0.10, and microblister scores were 2.6±0.25 and 2.3±0.34 for SKH-1 hairless and C57BL/6 mice, respectively (Fig 2 B and C, Table 2).

In both mouse strains, severe to moderate edema or erythema was observed by 72 h of NM exposure that decreased by 120 h (Fig. 1A and B iv-v, Fig 2 A–C, Tables 1 and 2). Furthermore, small and large microblister-like appearances with swelling, advanced to compressed skin with smaller microblister-like appearance by 72 h post-NM exposure in both SKH-1 hairless and C57BL/6 mice (Fig. 1A and B iv, Fig 2 A–C, Tables 1 and 2). By 120 h of NM exposure, there was a drastic decrease in microblister formation in both mouse strains but thick, leathery, wrinkled and damaged skin appeared at this time point (Fig. 1A and B v, Fig 2 A–C, Tables 1 and 2).

In SKH-1 hairless mice, defined pigmentation changes with red, yellow, whitish skin patches were also observed after 24 h of NM exposure. Conversely, this was less evident in C57BL/6 mice where small wounds and dry scaly skin patches were prominent at this time point (Fig. 1A and B iii, Fig 2 A, C and D, Tables 1 and 3). Wounded areas and pigmentation changes increased by 72 h and developed into desquamating skin areas and more distinct altered pigmentation by 120 h after NM exposure in both mouse strains. However, wounding was more prominent with bluish distinct spots in C57BL/6 mice when compared to SKH-1 hairless mice at both 72 and 120 h time points (Fig.1A and B iv-v, Fig 2 A, C and D, Tables 1 and 3). In SKH-1 hairless mice, patchy xerosis appeared by 72 h after NM exposure, but large areas of xerotic wrinkled skin were observed at this time point in C57BL/6 mice (Fig.1A and B iv, Fig 2 A, C and D, Tables 1 and 3). In contrast, by 120 h of NM exposure, leathery and wrinkled skin with desquamation was predominant in both mouse strains (Fig. 1A and B v, Fig 2 A, C and D, Tables 1 and 3). Related to these observations, NM-induced skin injury was greater with scores of 7.2±0.37 and 8.0±0.32 after 72 h, and 8.5±0.50 and 8.7±0.33 at 120 h after NM exposure in SKH-1 hairless and C57BL/6 mice, respectively (Fig. 2A, Table 1). Wounding and altered pigmentation were most evident at 120 h of NM exposure and were represented by scores of 4.5±0.30 and 4.6±0.33 in SKH-1 hairless and C57BL/6 mice, respectively (Fig. 2 D, Table 3). The xerotic desquamating skin, prominent at 120 h of NM exposure in both strains, is indicated by scores of 3.5±0.5 and 3.6±0.33 in SKH-1 hairless and C57BL/6 mice, respectively (Fig 2 E, Table 3). Also, as NM-induced skin injury progressed in both strains, mice appeared dehydrated and lethargic by 72 h, and suffered 40–60% mortality within 120 h after NM exposure.

#### Skin bi-fold thickness

**Figure 3:**
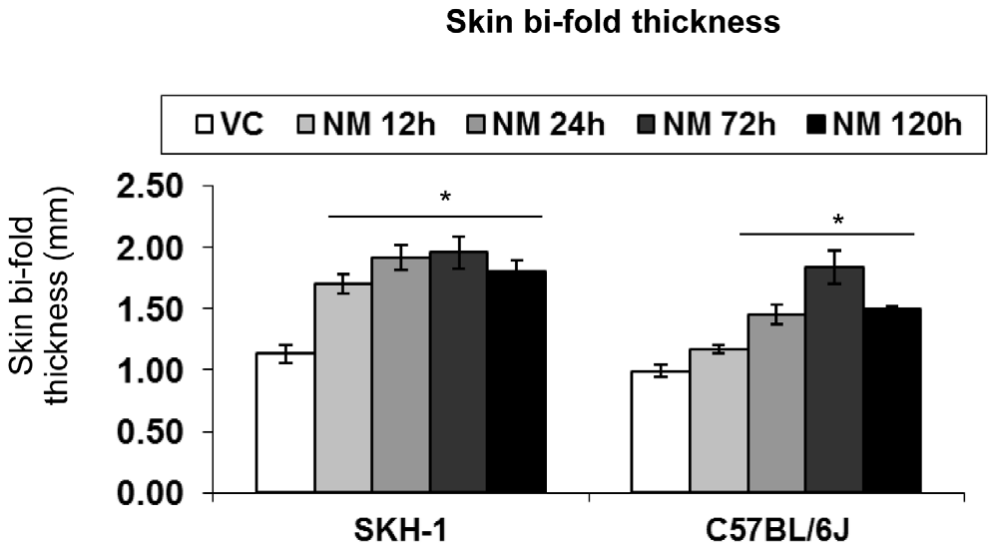
Effect of time-dependent NM exposure on the skin bi-fold thickness in SKH-1 hairless and C57BL/6 mice. Dorsal skin of mice was exposed topically to either 200 µL of acetone or NM (3.2 mg) in 200 µL acetone. Skin bi-fold thickness was measured as a function of time following NM exposure using a digital caliper as detailed under Materials and Methods. Data presented are mean ± SEM of 2–5 animals in each group. *, p<0.05 compared to respective vehicle control; VC, vehicle control.

Our studies with SM analog, 2-chloroethyl ethyl sulfide (CEES), have previously established skin bi-fold thickness as a useful biomarker that indicates skin edema [22], [32]. Since we had observed substantial skin edema at 12–120 h after NM exposure, we also measured skin bi-fold thickness at these time points. There was a significant (p<0.05) increase in skin bi-fold thickness in both strains following 12–24 h of NM exposure (Fig. 3). A more pronounced NM-induced increase in skin bi-fold thickness (1.5 and 1.7 fold versus control) was observed in SKH-1 mice early on (12 and 24 h after exposure; Fig. 3). In C57BL/6 mice, the greatest increase in NM-induced skin bi-fold thickness (2.0 fold) was noted at 72 h after exposure (Fig. 3). However, by 120 h after exposure, there was a 1.6 fold increase (versus control) in NM-induced skin bi-fold thickness in both strains (Fig. 3).

## Discussion

Appearance of SM cutaneous lesions, mostly studied in the Iranian population and veterans exposed to SM in Iran-Iraq war, could be categorized into acute and chronic phases [6]–[9]. In acute phase after SM exposure, erythema and edema with burning appears 2–24 h, followed by appearance of small vesicles within 18 h, which merge to form large bullae or blisters containing clear yellow fluid [6], [7], [11], [12], [31]. By 48 h post-exposure, larger blisters appear. These can burst, causing epidermal loss, ulceration or wounding, necrosis and/or eschar formation. These lesions can heal within a few weeks, but hyperpigmentation, hypopigmentation and scarring may remain [6], [7], [11], [12]. In the current study, 12–24 h of NM exposure also caused edema and erythema, microblister formation, and altered pigmentation in both mouse strains. However these were more prominent in SKH-1 hairless mice. Similar to reports in other animal models with SM and NM, we also observed only microblister formation and/or sub-epidermal blisters in both mouse strains; however, gross blisters that occur in humans, were not observed.

The chronic skin effects of SM include pruritis, xerosis (dry skin), altered pigmentation, scars, and angiomas [6], [7], [11], [12], [14], [15]. In the present study, NM exposure in both mouse strains also showed pigmentation changes, dry skin, and scars or wounding at later time points (72–120 h of exposure). However, wounding and dry skin appeared by 24 h of exposure in C57BL/6 mice, and darker bluish patches were also observed at 72 h of exposure suggesting slightly higher susceptibility of this strain to NM. Alternatively, this could have been an artifact due to shaving of hair in these mice, potentially causing dryness, bruising or other subtle wounding of skin. This caveat with C57BL/6 mice could be one of the reasons that SKH-1 hairless mice have most frequently been used to study toxic effects of SM and its analogs on skin tissue. Our studies along with others have also established useful biomarkers of skin injury in this mouse strain [32], [33], [35]–[39]. The current study also suggests that the SKH-1 hairless mouse could be a better model to study clinically relevant effects of NM, since the skin lesions of these mice were easier to observe and evaluate without the potential confounding effects due to the need for shaving in haired mice. Also importantly, NM-induced skin lesions in mice parallel clinical manifestations of skin injury due to SM in various animal models including mouse, pig, weanling pig and guinea pig, where delayed onset of symptoms including edema and erythema, microvesication, necrosis, ulceration, and altered pigmentation can occur [36], [40], [41]. NM application was carried out in acetone that could cause slight skin dryness or other effects; however, acetone alone does not show any significant effect on SKH-1 mouse skin as reported by us in a previous study [22].

The initial latency period and subsequent lesion development is related to the concentration, delivery, and duration of SM exposure, as well as temperature, humidity and other factors [7], [31], [42]. In mild exposures, skin lesions may be limited to erythema, and in 10–15 days, lead to altered pigmentation. However, in humans, moderate to severe exposures cause blister formation with healing within 2–3 weeks. In addition, skin erosions, with or without altered pigmentation, can occur [31], [42]. The clinical manifestations due to NM exposure observed in this study appear to parallel those due to moderate to severe dose of SM in humans. However, since NM exposure was carried out as its application in liquid form dissolved in acetone, there could be some variations in its injury development as compared to SM vapor exposure. The NM dose used in the present study is adequate to quantify effects of countermeasures on the progression of injury up to the time of death; a smaller NM dose depicting lesser injury could also be helpful in quantifying potential countermeasure effects on the healing process. Though mortality due to SM exposure is inconsistent, high level exposures, such as that used in the massacre of a Kurdish population in Iraq [6], could cause mortality due to skin injury, systemic absorption, secondary infection(s) and/or combined effects of other chemicals used during the attack. The observed NM-related 40–60% mortality in both the mouse strains observed in our present study suggests the possibility of dehydration, potentially related, at least in part, to excessive insensible water loss through the damaged skin. Poor water and/or food intake due to overwhelming systemic effects also may have contributed to mortality; further studies are needed to better characterize the systemic effects of topical NM exposure.

In summary, we identified clinically relevant biomarkers of skin injury progression with a SM analog NM, which evolved similarly in both haired and hairless mouse strains, and the lesions parallel those reported with SM in humans and other animals. These biomarkers of clinical effects on skin due to topical NM exposure could be useful in conducting accelerated laboratory screening of effective rescue therapies for skin injury by vesicating agents.
